# Sensitive Skin: Lessons From Transcriptomic Studies

**DOI:** 10.3389/fmed.2019.00115

**Published:** 2019-05-28

**Authors:** Adeline Bataille, Christelle Le Gall-Ianotto, Emmanuelle Genin, Laurent Misery

**Affiliations:** ^1^LIEN, F-29200, Univ Brest, Brest, France; ^2^Department of Dermatology, University Hospital, Brest, France; ^3^UMR1078 “Génétique, Génomique Fonctionnelle et Biotechnologies”, INSERM, Univ Brest, Brest, France

**Keywords:** sensitive skin, transcriptomics, pathophysiology, pathogenic mechanism, microarray, RNAseq

## Abstract

In 2016, a special interest group from the International Forum for the Study of Itch defined sensitive skin (SS) as a syndrome that manifests with the occurrence of unpleasant sensations (stinging, burning, pain, pruritus, and tingling sensations) after stimuli that should not cause a reaction, such as water, cold, heat, or other physical and/or chemical factors. The pathophysiology of sensitive skin is still poorly understood, but the symptoms described suggest inflammation and peripheral innervation. Only two publications have focused on sensitive skin transcriptomics. In the first study, the authors performed a microarray comparison of SS and non-sensitive skin (NSS) samples and showed differences in the expression of numerous genes in SS and NSS samples. Moreover, in the SS samples, two clusters of genes were identified, including upregulated and downregulated genes, compared to NSS samples. These results provide some interesting clues for the understanding of the pathophysiology of SS. The second study compared SS and NSS samples using RNA-seq assays. This method allowed the identification of long non-coding RNAs (lncRNAs) and differentially expressed mRNAs and provided a comprehensive profile in subjects with SS. The results showed that a wide range of genes may be involved in the pathogenesis of SS and suggested pathways that could be associated with them. In this paper, we discuss these two studies in detail and show how transcriptomic studies can help understand the pathophysiology of sensitive skin. We call for new transcriptomic studies on larger populations to be conducted before putative pathogenic mechanisms can be detected and analyzed to achieve a better understanding of this complex condition.

## Definition of Sensitive Skin

In 2016, the definition of sensitive skin (SS) was established by a special interest group from the International Forum for the Study of Itch. SS is defined as a syndrome defined by the appearance of unpleasant sensations (stinging, burning, pain, pruritus, and tingling sensations) in response to stimuli that would not normally cause such sensations (1). SS is an essential topic because 50% of women and 30% of men in Europe consider themselves to have SS (2, 3). All of these symptoms induce real discomfort and are important reasons to study SS. The symptoms of SS may be induced by different factors, including physical factors (such as ultraviolet radiation or temperature), chemical factors (such as cosmetics or water), environmental factors (such as pollution), psychological factors (such as stress or emotions), or hormonal factors (such as the menstrual cycle) (4–6).

## Known Concepts on the Pathophysiology of SS

The pathophysiology of SS has not been completely elucidated. It is commonly viewed as a multifactorial skin disorder with multiple pathways potentially involved. Although the pathophysiology of SS remains unclear, the underlying direct mechanisms are not immunological or allergic. Several differences have been found between SS and non-sensitive skin (NSS) (3, 4, 7–9). Classically, SS syndrome is considered a consequence of sensory neural changes and/or a skin barrier disruption that increases the permeability of the stratum corneum, resulting in an increase in transepidermal water loss (TEWL) (10–12). However, this impairment in the cutaneous skin barrier is not always present (9). Some authors have also focused on the vascular reactivity and have described a higher vascular response in some SS patients (9, 13).

SS is also characterized by sensory hyperreactivity since sensory symptoms have been reported in SS patients, suggesting a neurosensory dysfunction of nerves in the skin. Recently, Buhé et al. showed an alteration of the Aδ or C fiber populations with a significant decrease in fiber number/mm at the dermo-epidermal junction (14). This result was strengthened by a recent study showing that SS could be a small fiber neuropathy using quantitative sensory testing (15).

To date, we have to consider that the pathophysiology of SS seems to be related to a multiplicity of factors and therefore to multiple potential pathways. Consequently, some authors have focused on transcriptomic studies to find new lines of research to elucidate the mechanisms underlying SS syndrome.

## Contribution of Transcriptomics in a Physiopathology Study

Transcriptomics consists of the analysis of the transcriptome by generating genome-wide mRNA profiles, allowing a global description of gene expression under specific conditions, mainly using DNA microarrays (chips) or next-generation sequencing technologies (RNA-seq) (16, 17). These two methods enable the establishment of gene expression profiles to detect differentially expressed genes between healthy and pathological tissues. Microarrays are generally designed to profile the expression levels of known genes (18). For RNA-seq, it is possible to profile known genes and to discover new genes and gene variants (splicing isoforms) (18). Several teams have carried out comparative studies of these two platforms. Advantages and disadvantages have been found for each of them. It is clear that RNA-seq allows the discovery of a larger number of differentially expressed genes than microarrays. However, it has been notably demonstrated that both methods can highlight many differentially expressed genes that are unique to each platform (19).

## Lessons from Transcriptomic Studies of SS

To our knowledge, only 2 transcriptomic studies have been performed for SS. The first study was carried out by a Korean research team using microarrays (16). Eighteen individuals (9 SS and 9 NSS) had two skin biopsies performed following either lactic acid or normal saline application. For the microarray experiments, samples were pooled by groups of 3 so that 12 microarrays experiments were performed (3 for SS with lactic acid application, 3 for SS with normal saline application, 3 for NSS with lactic acid and 3 for NSS with normal saline application). The second study was performed by a Chinese research team using RNA-seq on, respectively 3 SS and 3 NSS samples (17). Taken together, the results of these 2 studies showed that a large number of genes were differentially expressed between SS and NSS samples. Indeed, Yang et al. showed that a total of 33 and 950 long non-coding RNAs (lncRNAs) and messenger RNAs (mRNAs) were upregulated in SS, and a total of 38 and 1565 lncRNAs and mRNAs were downregulated (17). Kim et al. found that in SS biopsies, 17 genes were upregulated and 29 were downregulated (16).

As specified earlier, the two techniques do not have the same ability to generate results and it is thus difficult to compare results between microarrays and RNA-seq. Moreover, in their publication, Yang et al. (17) only provided the list of the top 20 most differentiated genes between SS and NSS and no information on the other genes. It is therefore impossible to compare the differential expression of genes between the two publications.

More than mRNAs, the RNA-seq platform allowed the identification of light lncRNAs, which are a class of RNA transcripts more than 200 nucleotides long that are not translated into proteins. However, these RNAs are very important because they can play different functional and structural roles in many biological processes (20). Among the 20 lncRNAs upregulated or downregulated in SS described by Yang et al., few were known (17). This could be explained by the fact that lncRNAs have been little studied so far, especially in skin disorders. For example, lncRNA-*H19* was downregulated in SS. lncRNA-*H19* has been studied in the differentiation of keratinocytes where its role is to regulate the differentiation process via the miR-130b-3p/Desmoglein1 pathway (21).

### The Role of Innate Immunity

Although the underlying direct mechanisms are not immunological (3), many coding genes involved in specific inflammatory and immune responses are upregulated, such as *IGHA1/2* (immunoglobulin heavy constant alpha 1 and 2), *CDH1* (cadherin type I), *HLA-C* (major histocompatibility complex class I, C), *TLR1* (toll-like receptor 1), *S100A8* (S100 calcium binding protein A8) and the non-coding gene *GATA3-AS1* (16, 17). Thus, a role of the innate immune system is possible through the activation of PRRs (pattern recognition receptors), such as TLR1, whose mRNA is upregulated in SS (17). PRRs recognize structures conserved among species called PAMPs (pathogen-associated molecular patterns) as well as DAMPs (damage-associated molecular patterns) and induce the upregulation of gene transcription coding for proinflammatory cytokines such as IFNs (type I interferons), chemokines and antimicrobial proteins (22). S100A8 has been characterized as a DAMP and can interact with TLRs, specifically TLR4, to form heterodimers (S100A8/A9) (23, 24). Furthermore, Yang's team observed the expression of key cytokines and chemokine genes involved in inflammation, such as *IL27RA* (interleukin 27 receptor subunit alpha) and *CCL18* (C-C motif chemokine ligand 18) (17).

### The Role of Metabolic and Ion Transport/Ionic Balance Genes

Surprisingly, Kim et al. showed adiponectin deficiency in SS, which is known to be associated with dysfunctions in muscle contraction and metabolic homeostasis (16, 25, 26). Additionally, they identified many downregulated genes related to muscle composition/contraction, carbohydrate/lipid metabolism and ion transport/ionic balance. For instance, they demonstrated that ACVR1C (activin A receptor 1C), a type I serine/threonine kinase receptor for the TGF-β (transforming growth factor) superfamily, had a role in the pathogenesis of pain in SS (25). They highlighted that the knockdown of *ACVR1C* expression in human RD striated muscle cells, used as an *in vitro* model for SS, involved a Ca^2+^ dysregulation, which may be associated with a decrease in the pain threshold. They explained that this would result from the impairment of homeostasis and pain induction due to an increase in the expression of *TRPV1* (transient potential cation channel subfamily V member 1), *ASIC3* (acid-sensing ion channel 3) and the pain-related neurotransmitter *CGRP* (calcitonin gene-related peptide). Furthermore, the expression of *TRPV1* mRNA increased in SS vs. NSS, which has already been described by other studies (3, 27). Surprisingly, the qualitative analysis of the increased expression of TRPV1 proteins in SS did not demonstrate significant differences between SS and NSS (14, 27). However, in their study, Ehnis-Perez et al. showed that TRPV1 presence was less evident in patients who were unresponsive to the lactic acid test (27). Furthermore, the overactivation of TRPV1 based on the overexpression and/or hypersensitization of the receptor may influence the induction of SS (3, 27, 28).

### Keratinocytic Disorders

Kim's team showed an upregulation of the *CDH1* gene in SS (16). This gene encodes for E-cadherin, a transmembrane protein implicated in cell-cell adhesion that plays a key role in the maintenance of keratinocyte differentiation and epithelium tissue integrity (29). Furthermore, E-cadherin is involved in the PI3K-Akt signaling pathway, a pathway highlighted in Yang's publication that may play a key role in the pathogenesis of SS (17, 29, 30). Thus, PI3K-Akt signaling has been shown to be involved in the control of keratinocyte differentiation and/or the suppression of apoptosis (31). In addition, a repair mechanism may be induced that could explain the overexpression of a number of genes involved in the maintenance of epidemic homeostasis, including *KRT27* (keratin 27), *CLDN5* (claudin 5), *ANXA6* (Annexin XI), and *ERBB4* (epidermal growth factor receptor 4). *ANXA6* has been shown to be upregulated in both SS and atopic skin but downregulated in psoriatic skin (32). *ANXA6* encodes a calcium-dependent membrane protein that plays a role in keratinocyte differentiation. *ERBB4*, which was found to be increased in SS, is expressed in the epidermis in healthy human skin, and its deregulation affects keratinocyte proliferation and differentiation (33). These effects could disrupt the skin barrier described in some studies (9, 10, 34). In addition to the PI3K pathway involved in SS, other pathways, such as extracellular matrix (ECM) receptor interactions and focal adhesion, could play an important role in the pathogenesis of SS (17).

### The Roles of Sensory Neurons and Merkel Cells

The involvement of innervation has been increasingly described in the pathophysiology of SS. Among the differentially expressed genes identified in Yang's publication, some are known to be expressed either on peripheral or central neurons (17). The *DOCK9* (dedicator of cytokinesis 9) gene encodes a protein regulating the growth of dendrites in neurons (35). When *DOCK9* was downregulated in SS, a decrease in nerve fiber number was observed (14). The mRNA of the mechanosensitive ion channel *PIEZO2* has also been shown to be decreased in SS (17). This channel is expressed in sensory neurons and in the human skin and is involved in proprioception and touch (36). PIEZO2 is also the Merkel-cell mechanotransduction channel (37), and the genetic deletion of Merkel cells and associated mechanosensitive Piezo2 channels in the skin are sufficient to produce the conversion of touch to itch (38). Hence, PIEZO2 and Merkel cells could have an important role in SS pathophysiology.

## Conclusions

Transcriptomics gives a general signature of gene expression. In this study, we have summarized the results of the only two transcriptomic studies performed so far to compare SS and NSS samples. These two studies were conducted on rather small sample sizes: 3 pools of 3 individuals in each condition for the microarray study and 3 SS vs. 3 NSS individuals for the RNAseq study. They did not apply any multiple testing corrections but rather only ranked genes which expression was the most significantly changed between SS and NSS samples. These results are therefore questionable and studies on larger sample sizes are urgently needed. Nevertheless, in both studies, the authors performed some additional functional (Kim et al.) or bioinformatic (Yang et al.) studies on their top signals that give consistent results and could partly mitigate the initial weakness of the studies. Yang's team has demonstrated the dysregulation of transcription in certain signaling pathways, including PI3K-Akt, focal adhesion and ECM receptor interaction signaling (17). Kim's team highlighted dysfunctions in muscle contraction and metabolic homeostasis related to adiponectin deficiency. Some inflammatory and immune responses were also suggested (16, 17). Finally, the involvement of innervation and Merkel cells in the pathophysiology of SS has been shown.

Hence, SS appears to be a multifactorial skin disorder. This review presents a comprehensive analysis of SS transcriptomes, which may facilitate the identification of SS pathogenesis mechanisms and the development of potential targets for therapeutic strategies. As previously done with rosacea (39), these two studies provide very interesting data allowing, orientating and suggesting further research. Transcriptomic studies on larger populations are needed but these studies give key data to focus on some pathogenic mechanisms.

## Author Contributions

AB has made all the reviewing of the articles on Sensitive skin studies and wrote the paper. CL has red and corrected the manuscript. LM has supervised and corrected the final version of the manuscript. EG has corrected the final version of the manuscript.

### Conflict of Interest Statement

AB has links of interest with Beiersdorf, Clarins and Galderma. LM has links of interest with Beiersdorf, Bioderma, Clarins, Expanscience, Johnson & Johnson, Nestlé Skin Health, Pierre Fabre, Roche-Posay Solabia, and Uriage. CL has links of interest with Shiseido, Beiersdorf, Pierre Fabre and Clarins. The remaining author declares that the research was conducted in the absence of any commercial or financial relationships that could be construed as a potential conflict of interest.
